# “Tear down that wall”—a critical evaluation of bioinformatic resources available for lysin researchers

**DOI:** 10.1128/aem.02361-23

**Published:** 2024-06-06

**Authors:** Sophia Bałdysz, Robert Nawrot, Jakub Barylski

**Affiliations:** 1 Department of Molecular Virology, Institute of Experimental Biology, Adam Mickiewicz University, Poznań, Poland; Unversidad de los Andes, Bogotá, Colombia

**Keywords:** lytic enzymes, virology, domains, bacteriophages

## Abstract

Lytic enzymes, or lysins for short, break down peptidoglycan and interrupt the continuity of the cell wall, which, in turn, causes osmotic lysis of the bacterium. Their ability to destroy bacteria from within makes them promising antimicrobial agents that can be used as alternatives or supplements to antibiotics. In this paper, we briefly summarize basic terms and concepts used to describe lysin sequences and delineate major lysin groups. More importantly, we describe the domain repertoire found in lysins and critically review bioinformatic tools or databases which are used in studies of these enzymes (with particular emphasis on the repositories of Hidden Markov models). Finally, we present a novel comprehensive, meticulously curated set of lysin-related family and domain models, sort them into clusters that reflect major families, and demonstrate that the selected models can be used to efficiently search for new lysins.

## INTRODUCTION

Lytic enzymes (often termed lysins) can be defined as proteins that specifically cleave peptidoglycan, which is the main component of the bacterial cell wall (1–3). Such cleavage often results in the breach of structural integrity of the cell and the osmotic lysis of the bacterium. This trait makes lytic enzymes promising candidates for novel antimicrobials. Hence, lysins have raised considerable interest in the scientific and medical community in the age of rampant antibiotic resistance and growing mortality rates caused by drug-resistant bacteria (4). A significant number of studies demonstrated the efficacy and safety of antimicrobials based on lytic enzymes in preclinical settings (5–7) and a few clinical trials (8–11). Moreover, lysins are thought to inflict less collateral damage on the patients microbiome because their activity is usually genus-, species-, or even strain-specific (12, 13).

Thus, many researchers believe that lysins may be one of the solutions to the antibiotic resistance problem that scientists have been looking for. Application of these enzymes may ease the ongoing combat with antibiotic-resistant bacteria in medicine, veterinary (14), food processing, and surface decontamination. Additionally, lytic enzymes may be used in pathogen detection or disruption of bacterial cells during protein or nucleic acid isolation (1, 15).

Such diversity of possible applications ensures a high demand for novel lytic enzymes. Fortunately, natural metagenomes seem to contain a virtually inexhaustible repository of novel, undiscovered lytic proteins (16). However, the extraction of useful proteins from the hodge-podge of genes and genomes is not a trivial task, and the immense diversity of lysins makes it even harder. These proteins come from many different gene families, folds, and evolutionary backgrounds, and the only feature that they all have in common is the ability to degrade the bacterial cell wall (17).

## LYTIC PROTEIN GROUPS

To date, five classes of lytic enzymes have been identified: eukaryotic lysozymes, bacterial autolysins, bacteriolysins, virion-associated lysins, and endolysins (Table 1).

**TABLE 1:** Classes of lytic enzymes

Lytic enzyme class	Origin	Description	Target bacteria
Eukaryotic lysozymes	Animal/plant/fungi chromosome	Found in secretions, cleaves the peptidoglycan in the bacterial cell wall	G+, weak inhibitory effect on G- (16)
Autolysins	Bacterial chromosome	Murein hydrolases, locally degrade the cell wall during its remodeling or division	G+, G−
Bacteriolysins	Bacterial chromosome/plasmid	Class IIIa protein bacteriocins, released by a G+ bacterium to kill competing microorganisms	Some G+ bacteria
Virion-associated lysins	Phage genome	Locally digest the cell wall at the start of infection to allow injection of the phage genetic material	G+, G−
Endolysins	Phage genome	Degrade the cell wall at the end of the replication cycle to release viral progeny	G+, G−

Lysozymes are defence proteins expressed by animals (18, 19), plants (20, 21), and fungi (12) to combat bacteria, which colonize their bodies or their natural habitat.

In this paper, we focus on four groups of lytic enzymes of bacterial and viral origin. The first group are autolysins—enzymes used by bacteria to remodel their cell wall during cell growth, division, programmed cell death, and peptidoglycan recycling (1, 3, 22, 23).

The second major type of lytic enzymes includes bacteriolysins. These proteins are classified as a subgroup of bacteriocins (namely class IIIa) and are used by microorganisms to inhibit potential competitors in the environment (24, 25). Known bacteriolysins, include lysostaphin, zoocin A, millericin B, enterolysin A. All of the mentioned polypeptides are encoded on plasmids or chromosomes of the members of the phylum *Firmicutes* and target strains related to bacteria, which produce them (1, 3).

As implied by the name, virion-associated lysins (VALs) are an integral component of phage particles. They enable penetration of the viral genome into the host cell by local digestion of the peptidoglycan, which creates a small opening in the cell wall (26). They are common in phages which infect both Gram positive and Gram negative bacteria.

Endolysins, on the other hand, degrade the peptidoglycan to release phage progeny at the end of the replication cycle. They are known for their quick mode of action and can cause disintegration of a bacterial cell within seconds (15). These lytic proteins are synthesized in the cytoplasm but act in the periplasmic space. There are several mechanisms through which endolysins gain access to the bacterial cell wall (15, 21), including intrinsic secretory signals and “signal-anchor-release” domains (13). Nevertheless, the most common pathway relies on the action of holins. These small proteins associate in the cell membrane, forming irregular holes so large, that intracellular enzymes may freely access the murein layer. Most commonly, genes encoding lysins and holins are located in the vicinity of each other and form a lysis cassette. Endolysins have been most intensively studied as potential novel therapeutics (27). They share partial sequence and structure similarity with some VALs (28), however; enzymes associated with virions tend to be bigger, and their domain architecture is more complex.

The classification of lytic proteins presented in Table 1 is based on biological function but ignores the molecular mechanism of their action. Based on the type of bond, which they cleave in the peptidoglycan layer, these enzymes can be divided into five categories (29, 30):

1. *N*-acetyl-β-d-muramidases (also termed lysozymes, EC 3.2.1.17), which cleave the β-1,4-glycosidic bond between *N*-acetylmuramic acid and *N*-acetylglucosamine;
2. *N*-acetyl-β-d-glucosaminidases (EC 3.2.1.52), which cleave the β-1,4-glycosidic bond between *N*-acetylglucosamine and *N*-acetylmuramic acid;
3. Lytic transglycosylases (EC 4.2.2.n1, 4.2.2.n2), which cleave the bonds between *N*-acetylmuramic acid and *N*-acetylglucosamine, but contrary to the two former types, do not require a water molecule to break the glycosidic bond because the reaction which they catalyze is intramolecular and results in the formation of a 1,6-anhydro ring at the end of the *N*-acetylmuramic acid residue;
4. *N*-acetylmuramoyl-l-alanine amidases (EC 3.5.1.28), which cleave the bond between *N*-acetylmuramic acid and the first peptide of the peptide bridge;
5. Endopeptidases (EC 3.4.13.22, 3.4.14.13, 3.4.17.14, 3.4.16.4, 3.4.17.13, 3.4.19.11, 3.4.21.12, 3.4.24.32, 3.4.24.75), which cleave the peptide bonds within the oligopeptides which cross-link the aminosugar strands or the interpeptide bridges, which connect these oligopeptides.

The aforementioned enzymological categories may occur in different proportions in all functional classes of lytic proteins (31). For example, among endolysins there seems to be a disproportionately high incidence of amidases (2) and all known bacteriolysins are lytic endopeptidases.

The enzymatic activity of each lytic protein is determined by the type of enzymatically active domain (EAD), which is an evolutionarily conserved part of the protein (2). Many lysins also contain a cell wall-binding domain (CBD), which docks the enzyme to a specific element of the bacterial wall (29, 32). EAD and CBD domains may be separated by a short, flexible linker composed of polar and charged amino acids (33). Some lysins, including virion-associated lysins, may also contain domains which carry other functions, e.g., anchoring to the viral particle (13, 22). This clear-cut delineation of domain types is deceptively simple, but each type comes in numerous varieties. Additionally, while lytic enzymes must, by definition, have at least one catalytic domain, the number and arrangement of other domain types may differ (13, 22, 23, 28, 30).

It has been proposed that the evolution of endolysins occurred through domain shuffling and coevolution of phage endolysins with autolysins of their respective hosts (24, 25, 34). It is possible that similar exchanges occurred between all functional groups.

This paper summarizes current knowledge on lytic protein domains as well as details of lysin domain composition. We discuss database resources, which are currently available for lysin proteins, as well as tools used for their discovery and analysis. Finally, we carefully select a minimal non-redundant set of protein domains and associated Markov models which is sufficient to recognize a vast majority of known endolysins.

## ENDOLYSIN DATABASES

To date, there are a few databases which are explicitly dedicated to lytic enzymes: Enzybase (35), GMEnzy (36), phiBIOTICS (37), and PhaLP (22). Other databases contain few lytic protein records, however; they are not strictly dedicated to such enzymes. Links to these databases, along with their short descriptions, have been provided in Table 2. It is worth mentioning that most of these databases are historical and no longer maintained, but since many of them were used in lysin studies, they need to be discussed in this review.

**TABLE 2:** Lytic enzyme databases

Database name	Description	Number and type of lytic protein records	Record type	Link	Date of publication, last update, accession	Reference
Enzybase	Repository for endolysin, autolysin, bacteriocin, and lysozyme sequences	2,039 protein sequences	Protein sequences	biotechlab.fudan.edu.cn/database/Enzybase/home.php	2012, 2016, May 2020	(36)
GMEnzy	Repository for genetically modified enzybiotics	186 protein sequences	Protein sequences	biotechlab.fudan.edu.cn/database/gmenzy	2013, 2014, May 2020	(37)
phiBIOTICS	Repository for bacterial and bacteriophage enzybiotics	21 protein sequences	Protein sequences	www.phibiotics.org/index.php	June 2012, 2012, June 2020	(38)
PhaLP	Repository for phage lytic proteins	17,487	Protein and coding sequences, Interpro domains	www.phalp.org	2021, unavailable since November 2023, November 2023	(31)
MEROPS	Repository for peptidases and proteins which inhibit them	7 families (search words: name: “lysin” and substrate: “peptidoglycan”)	Protein family sequence alignments	www.ebi.ac.uk/merops/index.shtml	1996, September 2023, June 2020	(39)

Enzybase is a repository for endolysin, bacteriocin, autolysin, and lysozyme sequences, selected manually from the UniProt/Swiss-Prot database and scientific literature. At the time of publication Enzybase contained 1,144 enzybiotics from 216 natural sources (35), as of November 2023, this number has increased up to 2,039 sequences. A majority of the records in the database represent naturally occurring proteins, but some genetically modified enzymes are also included. According to the database authors, the sequences in this database contain 55 different InterPro domain types (35).

A sister database of Enzybase is GMEnzy—a repository of genetically modified enzybiotics (GMEs), including those obtained by domain shuffling and mutagenesis (36). Unfortunately, the number of records in GMEnzy has not increased over the last 8 years suggesting lack of further maintenance.

The third repository for lytic proteins is the phiBIOTICS database, which contains manually curated enzybiotics with confirmed therapeutic use. As in the case of Enzybase, the authors defined enzybiotics as all enzymes which cause microbial cell death, including endolysins, bacteriocins, autolysins, and lysozymes, regardless of their taxonomic origin (37). Unfortunately, this database contains only 21 sequences, all of which can be found in Enzybase.

The PhaLP database is a repository for phage lytic proteins: VALs and endolysins. The proteins, which are deposited within, were queried and selected from the UniProt database. However, a detailed description of the search process, as well as protein selection, is not described in the paper by the authors of the database. From these proteins, 1,604 VALs and 2,492 endolysins were manually curated (22). The authors of the database had annotated putative domains which can be found in these proteins and classified them as either VALs and endolysins. The last published version of the database includes 12,718 endolysins and 4,768 VALs as well as 26 EADs, 13 CBDs, and 66 miscellaneous domains which together compose 151 distinct lysin architectures. Regrettably, the database has been unavailable since early November 2023.

Additionally, several general purpose sequence databases contain data on lytic proteins. These collections include MEROPS, which is a peptidase database that includes lytic peptidases and UniProt—one of the biggest databases of protein sequences and annotations. The latter returns 15,493 records for a “autolysin AND (taxonomy_id:2)[Bacteria]” query, 7,942 for “endolysin AND (taxonomy_id:10239)[Viruses],” 22 for “bacteriolysin AND (taxonomy_id:2)[Bacteria].” The results of these queries likely underestimate the total number of lysin records in the database due to inconsistent nomenclature. On the other hand, this data set is redundant with some enzyme groups highly oversampled compared to others.

Additionally, lysin families or conserved domains which are building blocks of these enzymes are represented in databases of multiple sequence alignments (MSA) and Hidden Markov Model (HMM). The latter representation is important in our subsequent analyses since profile HMMs can be used to sensitively detect even very divergent members of protein families. Family and domain databases are summarized in Table 3.

**TABLE 3:** Hidden Markov Model databases

Database name	Description	Link	Database version used in the analysis	Reference(s)
PFAM	Database of protein families and domains	pfam.xfam.org	Version 33.0	(40)
Eggnog^*a*^	Contains data on orthologous relationships, functional annotation, and gene evolution	eggnog5.embl.de/#/app/home	Version 5.0	(41)
pVOGS	Contains data on orthologous groups of viruses which infect bacteria and archaea	ftp.ncbi.nlm.nih.gov/pub/kristensen/pVOGs/home.html	No longer accessible, accessed 01.02.2020	(42)
vogdb	Contains data on viral orthologous groups based on all RefSeq virus genomes	vogdb.org	Version vog99	(43–45)

^*a*^ The Eggnog database contains data on other data, not only viral and bacterial, however; in this work, we have used only a fraction of the Eggnog database, which contains only viral and bacterial domain and family data.

## EXISTING LYTIC PROTEIN SEARCH STRATEGIES

The past decades have seen a vast increase in the quantity of bacterial and phage genomes. Unfortunately, the annotation and experimental confirmation of protein functions lags far behind. This prompted several research groups to develop bioinformatic methods and tools, which would identify lytic enzymes.

Previously developed methods for lytic protein detection are summarized in Table 4. To the best of our knowledge, the first of such methods was developed by Ding et al. This approach allowed the detection of lysins based on their pseudo amino acid composition (PseAAC) (38) using stepwise Fisher’s Linear Discriminant Analysis or a support vector machine (SVM) as a classifier. Both estimators were trained on a small set of proteins, including autolysins and endolysins extracted from the Swiss-Prot database as positive examples (*n* = 42) and selected phage proteins. These proteins, incorrectly described as enzymes in the original paper, had less than 40% similarity as a negative set (*n* = 70) (39). Regrettably, the authors failed to publish a program or adequately describe the implementation. Nevertheless, the method was further developed, and subsequent works resulted in the publication of the Lypred tool, using a similar representation of protein sequences (PseAAC) with an improved SVM performance due to feature selection and training on an extended data set (40). This training set included 68 lysins and 307 non-lysin proteins (misnamed by authors as “lyases”) from phages and bacteria. The tool was initially released as a publicly available web-server but is no longer functional. The same fate befell two very similar machine learning (ML) methods that were developed by another group and trained on the modified Lypred data set. Both of them—CWLy-SVM (41) and CWLy-pred (42)—were based on protein sequence descriptors developed by Wei et al. (43). These descriptors are based on two distinct parts. The first was based on amino acids and dipeptides conserved among the represented protein and its putative homologs. The search for these homologs was performed against the nrdb90 database using PSI-BLAST. The results were merged with the query into a single consensus sequence, which was further transformed into a vector of amino acid and dipeptide frequencies. The second part of the features was based on the secondary structure predicted for the represented protein by PSIPRED and denoted content and distribution of helices, strands, or coils along the sequence. Both tools used the same implementation of an SVM classifier and a similar feature selection method. The last ML approach trained based on the modified Lypred data set—CWLy-RF used a random forest classification method, which was based on k-spaced amino acid group pairs (CKSAAGP). Unfortunately, this program was never publicly released.

**TABLE 4:** Tools used in lysin identification

Program type	Lysin detection method	Program example	Year of publication and accessibility^*a*^	Link
Machine-learning based	Pseudo amino acid composition of protein sequence	(39) Lypred	2009, inaccessible 2016, accessible	No link http://lin-group.cn/server/Lypred/
Machine-learning based	Conservation level of different amino acids based on calculated k-mers and secondary structure	CWLy-pred CWLy-SVM CWLy-RF	2020, inaccessible 2020, inaccessible 2021, inaccessible	http://server.malab.cn/CWLy-pred/index.jsp http://server.malab.cn/CWLy-SVM/index.jsp. No link
HMMER-based	Similarity to lysin sequence model	PhiBiScan	2013, accessible	http://www.phibiotics.org/index.php?nav=tools

^*a*^ In the case of inaccessible tools, the last accession attempt was made in September 2023.

Thus, according to our knowledge, the only operational lysin prediction software is the PhiBiScan tool, authored by the research group which developed the phiBIOTICS database. This pipeline is based on a HMMER program that scans query sequences for similarity against 16 Hidden Markov Models (HMMs) extracted from the PFAM database. These HMMs represent protein families and domains, which are thought to be connected with cell wall lytic activity based on literature data and database annotations (37).

Of course, one may search for lysin-related domains or protein families using HMMER3 directly, but this requires careful curation of the model collection used to recognize lytic proteins and calibration of an appropriate score or *e*-value threshold. These are precisely the issues with which we deal with in the last section of our paper.

Currently, one must conclude that the mentioned tools were not properly tested and validated. PhiBiScan was never rigorously benchmarked on a comprehensive set of test sequences. The machine-learning approaches, on the other hand, were tested during their development. Yet, while authors claim that their algorithms achieved excellent performance (sensitivity in the 0.67–0.95 range and specificity between 0.89 and 0.98), all reported tests were performed on small and poorly annotated data sets (41, 42, 44). Moreover, the used validation methods [jackknife and 10-fold cross validation (41, 42, 44)] do not exclude the possibility of overfitting due to the sparse, biased data set with significant internal redundancy. Finally, since none of the tools other than PhiBiScan are still available, the reported performance is not reproducible and should be interpreted with caution.

## LYSIN-RELATED DOMAIN AND FAMILY MARKOV MODELS

Domain and family databases have evolved rapidly over the last years. However, PhiBiScan—the only functional tool for the identification of lytic enzymes that remains publicly available—was developed over a decade ago. Thus, we have decided to revisit the problem of lysin detection and select a comprehensive set of lysin-related enzymes, as well as domains and families.

Hence, we first ran an HMMER search against the de-replicated set of bona-fide lysins from Enzybase (abbreviated further as Lysin90). The search returned a total of 225 models, which showed similarity to lytic enzymes. One hundred eighteen models were found to be significantly overrepresented in the Lysin90 sequences in comparison to the reference set composed of bacterial and phage records of UniRef90. The summary of the search and selection procedure is shown in Table 5.

**TABLE 5:** HMMER search results

Database name	No. of aligned Lysin90 proteins	No. of aligned UniRef90 proteins	Models aligned to Lysin90	Models statistically significant in the student (*t*-test)
PFAM	444	139,828	39	25
Vegg	173	25,830	15	8
Begg	428	105,520	78	30
vogdb	348	77,202	55	31
pVOGS	279	54,668	38	24

We found that only 13 models (presented in Table 6) are required to capture the nearly complete diversity of the Lysin90 proteins. These HMMs are mostly domains from the PFAM database (9), but families from begg (2) and pVOGS (2) were also included. They represent all major types of catalytic domains (including lysozymes, glucosaminidases, transglycosylases, amidases, and endopeptidases) as well as some binding ones (e.g., SH3 or LysM motifs). The description of these 13 representative models is shown in Table 6.

**TABLE 6:** Representative models in lytic enzymes

No.	Family and domain model	Description	No. of aligned Lysin90 proteins	No. of aligned UniRef90 proteins	Interproscan domain ID
1	PFAM:Lys	C-type lysozyme/alpha-lactalbumin family	25	80	IPR001916
2	pVOGS:VOG4649	Lysin; putative lysin; putative endolysin; hypothetical protein; ORF44; gp56; orf28; tail lysin; endolysin	52	202	
3	PFAM:SH3_5	Bacterial SH3 domain	38	182	IPR003646
4	begg:COG5632	*N*-acetylmuramoyl-l-alanine amidase	117	2,728	
5	PFAM:Glyco_hydro_25	Glycosyl hydrolases family 25	63	1,658	IPR002053
6	PFAM:Peptidase_M15_4	d-alanyl-d-alanine carboxypeptidase	51	1,726	IPR039561
7	PFAM:CHAP	CHAP domain	85	3,943	IPR007921
8	PFAM:Glucosaminidase	Mannosyl-glycoprotein endo-beta-*N*-acetylglucosaminidase	56	4,243	IPR002901
9	begg:COG5263	Repeat protein	30	2,779	
10	PFAM:Amidase_3	*N*-acetylmuramoyl-l-alanine amidase	27	10,075	IPR002508
11	PFAM:LysM	LysM domain	60	24,280	IPR018392
12	PFAM:Peptidase_M23	Peptidase family M23	43	28,590	IPR016047
13	pVOGS:VOG3721	Virion structural protein; putative tail fiber protein; putative murein lytic transglycosylase; glycoside hydrolase family protein; ORF181; putative phage-related lysozyme domain; putative tail fiber protein; tail fiber protein; putative soluble lytic; murein transglycosylase; putative phage tail	5	3,202	

The 13 HMMs may be sufficient to recognize almost all lysins in Enzybase, but it would be naive to expect that this database (consisting of only 2,039 well studied, therapeutically relevant enzymes) really covers the whole spectrum of lytic proteins. Bearing that in mind we performed an additional search to reveal any other lysin-like domains and families, which might have been missed during the initial analysis and highlight the relationships between different models.

**Fig 1:**
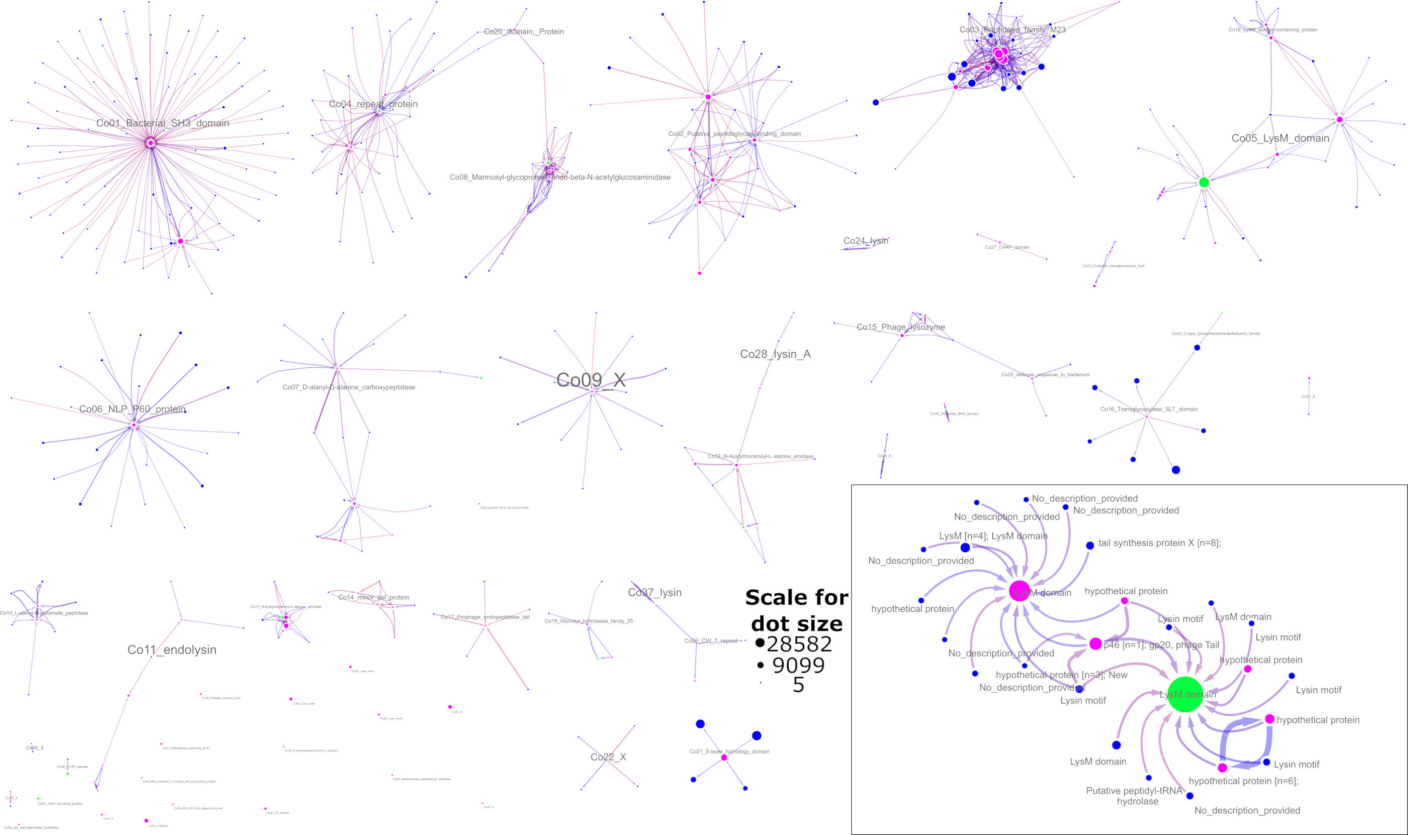
Model clusters with at least 75% jaccard containment. Green circles—minimal representative model set, pink—models significantly overrepresented amongst lytic proteins, blue—other, similar HMMs. The size of the marker corresponds to the number of proteins aligned to the model in the Uniref90 set. The edge colors are based on the standard deviation of the fraction of mutual overlap of domains (red indicates a higher fraction value). The opaqueness of every directed edge is proportional to the jaccard containment of the source domain in the target one, and its width is proportional to the jaccard index (both measured at the amino acid/protein position level). The direction of the edge reflects the jaccard containment value. Each arrow points from the embedded model to the encompassing one. If model alignments have significant overlap in both directions (match to similar sequences), then they are connected by two antiparallel edges. The panel on the lower right corner shows a close-up of two example clusters.

We compared the entire set of 118 models overrepresented in the lysin set to all models from PFAM, Vegg, Begg, vogdb, and pVOGS in terms of their co-occurrence in proteins and HMM-HMM similarity. The results from the co-occurrence analysis are shown in Fig. 1. Briefly, we found 421 lysin-like models grouped into 37 clusters and 17 singletons.

Generally, the functional annotations within clusters were consistent and, with caution, can be propagated to the related models with no annotations (mostly begg HMMs). A few clusters grouped functionally heterogeneous proteins. For example, a subgraph dominated by peptidases from the M23 family also included models described as lysozymes, endolysins, and tail proteins. This discrepancy may be the result of a spurious alignment but most likely is evidence of model missannotation.

**Fig 2:**
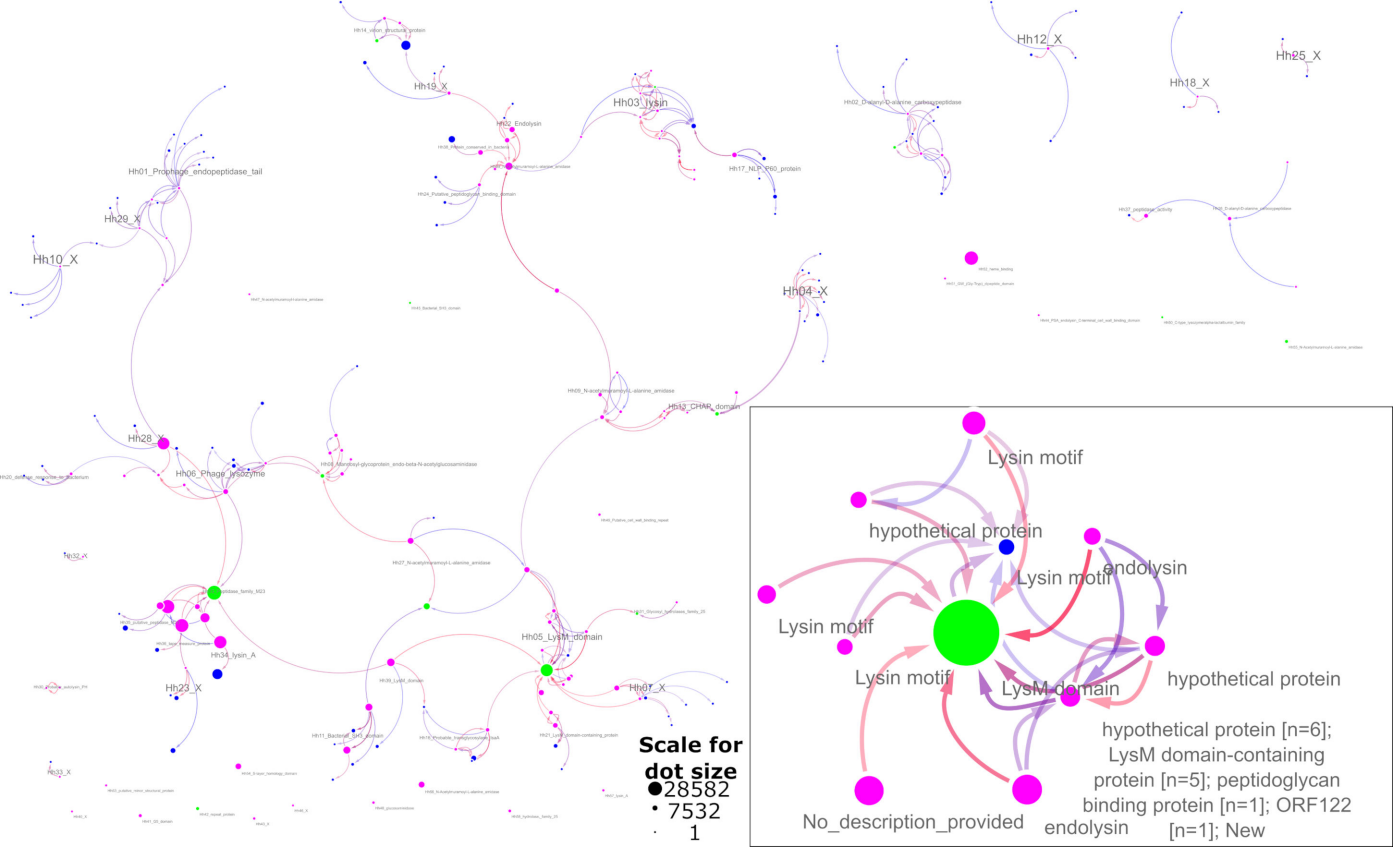
Model clusters from the HHsuite analysis. Green circles—minimal representative model set, pink—models significantly overrepresented among lytic proteins, blue—other, similar HMMs. The size of the marker corresponds to the number of proteins aligned to the model in the Uniref90 set. The edge colors are based on the probability that a model is similar (red indicates a higher probability). The direction of the edge reflects the jaccard index value. Each arrow points from the embedded model to the encompassing one. If model alignments have significant overlap in both directions (match to similar sequences), then they are connected by two antiparallel edges. The edge opaqueness is proportional to the HHblits positive match probability. The panel on the lower right shows a close-up of an example cluster.

In contrast to the co-occurrence analysis, the HHsuite search (HMM-HMM alignment) returned a much smaller number (221) of models. Most of them were grouped into 39 clusters of related HMMs, but 19 remain unbound. The similarity network is shown in Fig. 2 (the original Cytoscape similarity networks for both co-occurrence and HMM-HMM comparison are attached as supplementary files Colocation_analysis.cys and HMM2HMM_hhblits.cys, respectively, on the dryad repository).

As in the case of the co-occurrence network, most clusters have relatively consistent functional annotation. Conversely to the co-occurrence network, we observed more inter-cluster connections in the HHblits-based graph. Nevertheless, 19 models (including COG5263, SH3_5, Lys, COG5632) were not included in the clusters. Six of these models were also singletons in the co-occurrence network. The logical interpretation is that these HMMs represent unique binding or enzymatic domains or distinct lysin families. Some of the clusters did not include any of the 13 minimal representative HMMs, as in the case of the cluster for prophage endopeptidase tail proteins.

**Fig 3:**
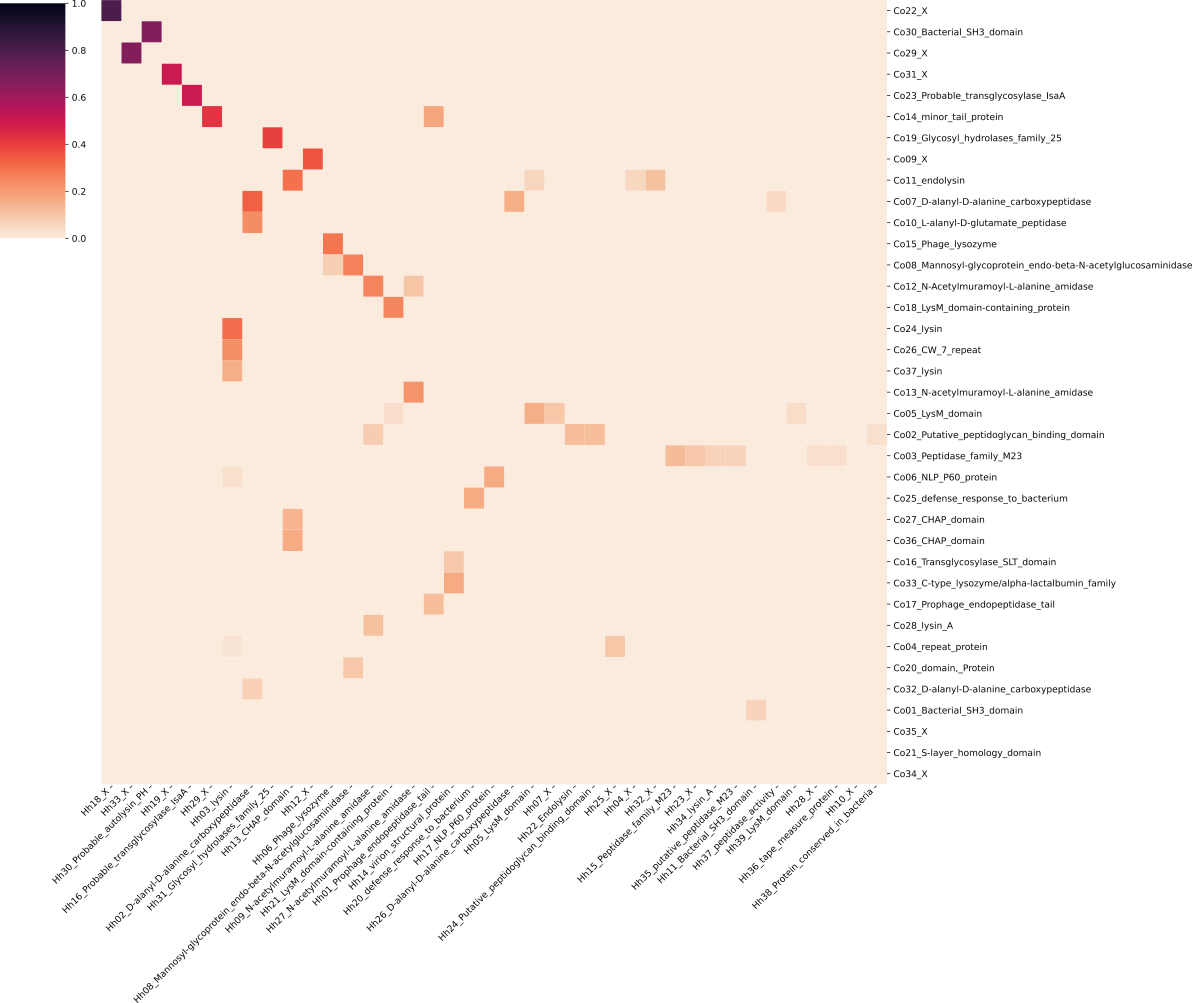
Similarity matrix of clusters from HHsuite analysis and co-occurrence. The color of the tile represents a Jaccard index of the corresponding clusters. Singletons are not shown for better readability of the matrix.

A comparison of the clusters obtained in both the co-occurence and HHsuite analysis (shown in Fig. 3) illustrates that these methods return somewhat different results. While a few clusters display high similarity (e.g., groups of models similar to SH_3 domain, NlpC/P60 family, and CHAP domain), many overlap partially with several clusters from the other method.

## COMPARISON OF OUR MODEL SET TO EXISTING TOOLS AND DATA SETS

A slightly similar work was earlier performed by the authors of the PhaLP repository. To construct the database, they extracted 45 PFAM, PROSITE, SMART, and CDD models (22, 45–47). Only 7 of these models were present in our minimal representative set, another 13 could be found in the set of 118 models overrepresented among EnzyBase lysins. It should be noted that these models were not selected to adequately cover all known types of lytic proteins, but rather, were selected manually to study the domain architecture of phage lysins alone (22).

We also compared our model sets to those applied in phiBiSCAN (37) and found that 6 of the 16 HMMs used in the tool were also a part of our minimal representative set. An additional 4 can be found among the 118 HMMs overrepresented among lytic proteins. This suggests that they are probably lysin-related but are not required to recognize enzymes from the Lysin90 set.

Two of the remaining models used by phiBiSCAN were identified in our co-location and HHsuite analyses. The first, SLT, can be found in numerous lytic transglycosylases and is likely a genuine lysin-related motif. Its omission in our analysis may be a result of the underrepresentation of proteins which contain this domain in Enzybase. The second, Peptidase_M15_3, occurs in sequences annotated as signaling proteins and polypeptides with no known function. This indicates that this model represents a functionally heterogeneous group of proteins and may not be suitable for specific detection of lysins. Finally, four of the models—Glyco_hydro_19, Hydrolase_2, Phage_lysis, and Peptidase_U40—did not appear in any of our sets. Hydrolase_2 can be found in cell wall hydrolases but also spore cortex-lytic enzymes. Glyco_hydro_19 occurs in various putative hydrolases, including chitinases and Phage_lysis can be found in Rz proteins and spanins. Such descriptions suggest that while some of the recognized proteins may be lysins, many of them represent functionally unrelated proteins. Finally, HMMER3 aligns the last remaining phiBiSCAN HMM—Peptidase_U4 to only one protein in the entire UniRef90 data set—LT_GEWL domain-containing protein, which indicates that the model might be outdated or faulty.

On the other hand, the phiBiSCAN models do not include seven models present in our minimal set, which may be necessary to detect some types of lysins. Hence, careful comparison of all model collections led us to believe that our minimal HMM set may be better suited to recognize *bona fide* lysins than the one used in the phiBiSCAN.

To confirm our assumptions, we tested the phiBiScan tool and the HMMER3 search results performed with our models on a benchmark set composed of the Lysin90 set and an equal number of non-lysin bacterial or phage records from UniRef90. For the minimum score threshold of 20, our model set reached a recall equal to 0.99 and a precision of 1.0, whereas phiBiScan with its default settings obtained 0.40 and 0.96, respectively. Thus, the F1 scores were 0.996 for our set and only 0.542 for phiBiScan. This indicates that the selected models can be used for specific detection of new instances of known endolysin families. All of the discussed models and sets formatted to use with HMMER3, HHsuite, and MMSeqs2 (the most popular domain and family search tools) together with application examples are available at the Dryad repository as “models_and_instructions.zip” file.

## IDENTIFICATION OF LYSINS FROM NOVEL PHAGE GENOMES AND METAGENOMIC DATA

To check whether the 13 selected HMMs can be used to identify novel lysins, we performed an HMMer3 search against proteins encoded in recently sequenced bacteriophage genomes. The aim of this experiment was to assess whether our proposed group of models can recognize previously unknown lytic enzymes, so we manually reviewed the NCBI nucleotide database for genomes published after the download of the last database used in this study (01.02.2023). To avoid overlap between the test set and our original databases, any sequence similar to a record published before the cut-off (more than 90% identity with at least 80% query coverage in a megablast search with default parameters) was excluded from the procedure. The only other requirement was that every analyzed genome had to include at least one gene annotated as a lytic enzyme to enable comparison of model hits to existing annotations. We compiled the genome set that included 10 phages of different genome sizes (18–79 kbp), taxonomic status (4 families, but mostly unclassified), and various hosts (including bacteria from phyla Pseudomonadota, Bacillota, and Actinomycetota, for details see Table S3). No genome or protein was added or removed from the test set after the HMM search to avoid skewing the results.

The initial result of the search for lysins in 10 recently sequenced bacteriophage genomes turned out to be somewhat discouraging. We identified only 6 of the 16 annotated lytic enzymes and found a single protein hit with no functional annotation (hypothetical protein of Staphylococcus phage PT1-9) (details in Table S3). However, after closer inspection, we discovered that some of the putative lysins are clearly missanotated. For two of the “false negatives,” we found no evidence of lysin status in InterProScan, CD-search, and BLASTp results or in original papers (if any). One of them displays high similarity to numerous dATP/dGTP diphosphohydrolase domain-containing proteins. The other protein has no blast hits, conserved domains, or available experimental evidence of the function. Moreover, three proteins that we initially included into the lytic enzyme set were labeled incorrectly due to ambiguous nomenclature (“glycoside hydrolase,” “glycosyltransferase,” and “tail fiber protein, peptidase domain”) and are not actually endolysins. Finally, the single protein that matched to our models but was not annotated as a lytic enzyme is similar to several known lysins, contains a number of lysin-associated domains, and is encoded near a holin gene (in the place in phage genomes where lysin genes tend to reside). Thus, if we take into account only the *bona fide* lysins, then the observed recall rises from merely 0.38 (6/16) to 0.58 (7/12) with perfect specificity. This metric is still not fully satisfactory, warranting the inclusion of additional models (e.g., the above-mentioned STL domain or Glyco_hydro_24 that we observed in the two missed lysins). Nevertheless, our results show that the current model set can capture the majority of novel lysins even in its present form.

Additionally, it may be worth mentioning that our approach enabled the identification of a new endolysin active against *Rothia* spp. (48) in metagenomic data. The activity of this enzyme has been proven by wet-lab experiments. This demonstrates that our models can be successfully used to find lysins in unannotated metagenomic data.

## SELECTION OF LYSIN-RELATED FAMILIES AND DOMAINS

To identify motifs connected with lytic activity, we selected non-genetically modified proteins from the Enzybase database as a comprehensive set of *bona fide* lytic enzymes (termed Lysin90). These sequences were de-replicated through MMSeq2 clustering to remove redundant records and reduce sampling bias connected with intensively studied phage and bacteria groups (e.g., lysins active against streptococci, enterobacteria or encoded by *Twortwirinae* phages). We chose a single representative from each cluster of sequences with at least 90% similarity and 80% coverage (compared to the longer sequence). As a result, the Lysin90 data set was reduced to 451 non-redundant proteins. The de-replication process mimicked the clustering procedure used in generating our “reference” group comprised of all bacterial and phage protein records from the UniRef90 database, downloaded as a paginated UniRef query filtered by the taxonomy ids for *Caudoviricetes* (taxonomy_id:2731619), Bacteria (taxonomy_id:2), or unclassified dsDNA phages (taxonomy_id:79205) and identity of 0.9.

To represent the analyzed domains and families, we used profile hidden Markov models (HMMs), which capture the consensus and variability within an analyzed sequence group and enable detection of orthologs of the represented sequences with more sensitivity than sequence-alignment tools, such as BLAST. The models were retrieved from the following databases: PFAM, pVOGS, vogdb, Eggnog (subdivided in further analysis into bacterial and viral sections, abbreviated as Begg and Vegg) (see Table 3 for database versions used in this article).

To identify the domains and protein families which are associated with endolysins, we used all aforementioned models to conduct the HMMER search against both Lysin90 (downloaded May 2020) and UniRef90 sets (downloaded January 2023) (with default settings). The results of both searches were filtered to remove hits with a score lower than 20 (corresponding to an *e*-value of ~1*e*−5 calculated for the smaller, Enzybase set) and those enveloped in other, higher-scoring hits (this procedure is often referred to as “culling” in relation to the “culling limit” of the BLAST tool). Then, domains and families, which are statistically overrepresented among lytic enzymes, were extracted by comparing the number of hits in the Lysin90 and UniRef90 data sets. We used the unpaired student *t*-test and selected results, with a *P*-value lower than 1*e*−5 and at least three hits in the Lysin90 set. Such a stringent filtering procedure was designed to avoid false positive findings, which might be the result of accidental database contamination or spurious alignments in the relatively small, positive data set.

The resulting set of profile HMMs over-represented in lytic enzymes was further refined to and a minimal set of most general models, which capture the diversity of the Lysin90 protein set, was selected. We performed random HMMs elimination by running a million replicates of the following procedure. First, a model was randomly dropped from the set unless its absence reduced the coverage of the Lysin90 data set (fraction of aligned proteins) below 98%. Then, the depletion step was repeated recursively until the exclusion of every model had been tested. Notably, only 443 of the 451 sequences in the Lysin90 data set (98.2%) had any hits to any of 118 *t*-test selected models at the beginning of the procedure. Using such an approach, we found that the minimal number of models, which cover at least 98% of Lysin90 proteins, is 13. However, the specified conditions are fulfilled by multiple 13-element combinations of 33 models. To find an optimal one, we ran an exhaustive search in the entire space of 5.73 × 10^8^ possible combinations and ranked them based on the coverage of the Lysin90 and the UniRef90 data set. The highest-ranking combination of models was selected and is presented in Table 6.

## MODEL COMPARISON AND SYNONYMS IDENTIFICATION

To elucidate the similarities between the lysin-associated and other, related models, we performed a comparison of domains and families selected by the *t*-test and all other records from the HMM databases. We performed an in-depth analysis of the co-location of HMMER3 hits within the UniRef90 protein set (selecting hits with a score not smaller than 20 with no out “culling” of overlapping domains) for 118 HMMs overrepresented in Lysin90 set and all other models from PFAM, Vegg, Begg, vogdb, and pVOGS database. For every pair of HMMs, we calculated the number of proteins with hits to both models, alignments of model A with which domain B overlaps by at least 1 amino acid and the total size of this overlap (number fraction of positions aligned to model A, which is covered by alignments of model B). These three numbers were then converted into corresponding jaccard containment indices, which indicate the fraction of proteins, alignments, and amino acids of domain/family A covered by a domain/family B (49, 50).

Additionally, we compared the analyzed models directly—through an HMM-HMM alignment with the HHblits tool from the HHsuite 3.3.0 (51).

Both positional (co-occurrence) and alignment-based (HHblits) comparisons were used to construct graphs of similar models. The first of these networks connected models with at least 75% jaccard containment for amino-acid positions, number of overlapping domains, and shared proteins. The second network connected models with HMM-HMM alignments from the HHblits tool (with a maximal *e*-value of 1*e*−5 and at least 75% template coverage) (51). Both graphs were visualized in the Cytoscape 3.9.1 environment, and the implementation of the Markov Clustering algorithm (MCL) provided by the clusterMaker plugin of this tool was used to delineate model clusters (with default settings). The obtained groups were compared based on the jaccard index of shared models.

Scripts used in all of the above analyses were written in Python 3.8 or Bash-5.2 and are available in the dedicated GitHub repository (https://github.com/zwmuam/lysin_vocabulary).

## COMPARISON TO EXISTING TOOLS

We compared the performance of the HMMER3 search with 13 representative lysin-related models and minimal score of 20 to the phiBiScan tool. Both methods were applied to search for lytic enzymes in the test set composed of Lysin90 set (451 sequences) and the equal number of randomly selected non-lysin bacterial or phage records from UniRef90 (available in the dedicated dryad repository and as the S1 and S2 files). The performance of both tools is reported as precision, recall, and F1 score.

## CONCLUSIONS

In this paper, we delineated major lysin groups and reviewed the databases and bioinformatic resources which can be used to identify novel enzymes in genomic and metagenomic data. We also described a comprehensive, meticulously curated set of lysin-related protein families and domains and sorted them into clusters of related motifs.

As presented above, merely 13 Markov models are required to adequately explain the diversity of lysins from EnzyBase. This is, however, a minimal HMM set that covers only well-annotated enzymes. Thus, these models may be used to perform a strict, conservative search for new members of known lysin families but may fail to recognize unrelated or highly diverged enzymes. In analyses which require higher sensitivity, but a certain number of false positives can be tolerated, expanding the model base (e.g., addition of an SLT domain) may be advisable. Our analysis provides a convenient framework for rational selection of the additional models by clustering HMMs into similarity groups. Hence, one may compile a custom set of models suitable for a specific purpose and use it to search a data set at hand with well-established bioinformatic tools like HMMer3, HHsuite, and MMSeqs2.

Moreover, our results shed new light on the lysin diversity by revealing similarities between different family and domain representations. Hence, if one considers that the domain architecture determines both the activity range and kinetic properties of the enzyme (52, 53), our results may also assist in the engineering of novel synthetic lysins with desirable characteristics.

## Data Availability

The source code used in this paper is publicly available at the dedicated GitHub repository (https://github.com/zwmuam/lysin_vocabulary).

## SUPPLEMENTAL MATERIAL

File S1 aem.02361-23-s0001.txt Protein sequences used in the analysis described in the paper.

File S2 aem.02361-23-s0002.xlsx Proteins from the Lysin90 dataset, with accession numbers, description, and associated model.

File S3 aem.02361-23-s0003.xlsx Sequences from phage genomes, including annotated lytic proteins as well as the accession numbers of novel phages used in the study.

ASM does not own the copyrights to Supplemental Material that may be linked to, or accessed through, an article. The authors have granted ASM a non-exclusive, world-wide license to publish the Supplemental Material files. Please contact the corresponding author directly for reuse.
